# On the Possibility of Using 3D Printed Polymer Models for Modal Tests on Shaking Tables: Linking Material Properties Investigations, Field Experiments, Shaking Table Tests, and FEM Modeling

**DOI:** 10.3390/ma16041471

**Published:** 2023-02-09

**Authors:** Pawel Boron, Jaroslaw Chelmecki, Joanna Maria Dulinska, Nadzieja Jurkowska, Bartlomiej Ratajewicz, Piotr Stecz, Tadeusz Tatara

**Affiliations:** 1 Faculty of Civil Engineering, Cracow University of Technology, 31-155 Cracow, Poland; 2 Polteron Engineering Co., Ltd., 30-709 Cracow, Poland

**Keywords:** shaking table tests, in-situ investigation of concrete chimney, 3D printing, PLA, modal analysis, 3D printed small-scale model

## Abstract

In this article, the possibility and the pertinence of using 3D printed polymeric materials for models in modal tests on shaking tables were recognized. Four stages of the research have been linked: The material properties investigation, the field experiment on the modal properties of the reinforced concrete chimney (a prototype), the shaking table tests on the modal properties of the 3D printed polymer model of the chimney, scaled according to the similarity criteria, and the numerical calculations of the FE model of the 3D printed mockup. First, the investigation of the properties of 3D printed polymer materials revealed that the direction of lamination had no significant effect on the modulus of elasticity of the material. This is a great benefit, especially when printing models of tall structures, such as chimneys, which for technical reasons could only be printed in a spiral manner with the horizontal direction of lamination. The investigation also proved that the yield strength depended on the direction of the lamination of the specimens. Next, the natural frequencies of the chimney, assessed through the field experiment and the shaking table tests were compared and showed good compatibility. This is a substantial argument demonstrating the pertinence of using 3D printed polymer materials to create models for shaking table tests. Finally, the finite element model of the 3D printed polymer mockup was completed. Modal properties obtained numerically and obtained from the shaking table test also indicated good agreement. The presented study may be supportive in answering the question of whether traditional models (made of the same material as prototypes) used in shaking table tests are still the best solution, or whether innovative 3D printed polymer models can be a better choice, in regard to the assessment of the modal properties and the dynamic performance of structures.

## 1. Introduction

The 3D printing technology, which has been developed for over 30 years, has found practical application in the construction industry. Several manufacturing technologies can be used in the process of 3D printing. The most common additive manufacturing techniques are as follows: Stereolithography (SLA), selective laser sintering (SLS), fused deposition modeling (FDM) or PolyJet [1,2]. Each technique can differ in material selection, surface finish, durability, and manufacturing speed and cost. The attractiveness of using 3D printing technology is also supported by the variety of materials, from which elements can be made of. Some popular materials used in 3D printing are acrylonitrile butadiene styrene (ABS), polylactic acid (PLA), nylon, poly(dodecano-12-lactam) (PA12), as well as concrete or metal [1,2]. The authors of scientific works on the use of 3D printing technology in civil engineering present the use of this method both during architectural design and at the stage of erecting buildings [3,4,5]. In particular, many works related to 3D printing of elements or entire buildings made of concrete and reinforced concrete should be noted here [6,7,8]. The authors develop new technologies for printing concrete objects, allowing for the creation of complex shapes of concrete surfaces, which are characterized by the good print quality and geometric accuracy [9,10]. In recent years, some studies have been conducted on the modal properties of structures made in 3D printing technology. The authors determine natural frequencies and modes of vibrations of simple structures, such as a cantilever beam [11] or a complex innovative metastructure [12] in theoretical, numerical, and experimental ways.

At present, shaking table tests on modal properties of structures are widely used in earthquake engineering. The results of shaking table tests are sometimes verified using field testing after the structure is completed. For example, the evaluation of the dynamic characteristics of tall buildings, using data from field experiments and from shaking table tests on mockups, was carried out for the Shanghai Tower (632 m high) [13] and the Famen Temple (147 m high) located in Shaanxi Province, China [14]. Then, in work [15], the modal properties obtained from the field experiment on a 33-story reinforced concrete building were compared to those obtained from the shaking table test on a 1/25-scale model, fabricated using fine-aggregate concrete reinforced with thin steel wires. Finally, the authors of the work [16] estimated the modal properties of the Sutong Bridge, which has the second longest main span (1088 m) of cable-stayed bridges worldwide, through in-situ experiments and through shaking table tests on a 1/35-scale model. The comparison of the results of modal analyses, obtained from the shaking table tests and the full-scale experiment, shows how useful dynamic similarity testing is with the use of the shaking table.

In the last decade of research, it has been proven that 3D printing technology can also be useful in shaking table tests. The authors of the work [17] tested the modal properties of a free-form concrete structure 3.6 m (length) × 3.0 m (width) × 2.5 m (height), imitating the Meiso no Mori municipal federal hall in Japan. The small-size model, satisfying the similitude laws, consisted of three brass bars 400 mm long and a free-form roof fabricated by a 3D printer. Similarly, the spire of the church of St. Mary Magdalene in Waltham-on-the-Wolds, UK, which was damaged in the 2008 Lincolnshire earthquake, was modeled for modal tests on a shaking table [18]. The majority of the spire was printed from powder using a ZCorp ZPrinter 350. Finally, an experimental study of modal properties and seismic response of a groin vault model was conducted on the shaking table at the University of Bristol, UK [19]. The 3D printed model was made of polylactic acid, which is a completely compostable and biodegradable polymer obtained from the processing of plants rich in dextrose. All the above research was an inspiration for the authors of this work to assess the possibility of introducing 3D printing technology to modal tests on shaking tables.

The main objective of this paper is to recognize the possibilities and the pertinence of using 3D printed polymeric materials for models in modal tests on shaking tables. Once this objective is accomplished, some advantages and disadvantages of implementing 3D printing technology to create models for shaking table tests can be identified. Finally, it will be possible to answer the question of whether traditional shaking table models, made of the same material as the prototypes, are still the best solution, or whether innovative 3D printed polymer models are a better choice in the context of the appropriate assessment of the modal properties of structures.

The novelty of the current paper lies in providing comprehensive and multifaceted studies that simultaneously cover four different research areas:

- The investigation of material properties of a 3D printed polymer material in the context of the rasterization;
- The field experiment on modal properties of the prototype structure, i.e., the reinforced concrete chimney 120 m high;
- The shaking table test on modal properties of the mockup of the chimney made of the 3D printed polymer material;
- The numerical investigation on modal properties of the 3D printed polymer model.

As shown in the literature review above, some studies concern 3D printed material properties, and some researches address the comparative analysis of the modal properties of a prototype and a 3D printed polymer model for shaking table tests; however, not many works focus on combining the results of the shaking table tests with numerical investigations on 3D printed models. However, linking the four above-mentioned types of research, covering all the major aspects of using 3D printed polymer models for shaking table purposes, is rarely, if ever, addressed in the available literature. This link makes this study original and innovative in the field of civil engineering.

## 2. Materials and Methods

### 2.1. The General Concept of the Research

**Figure 1:**
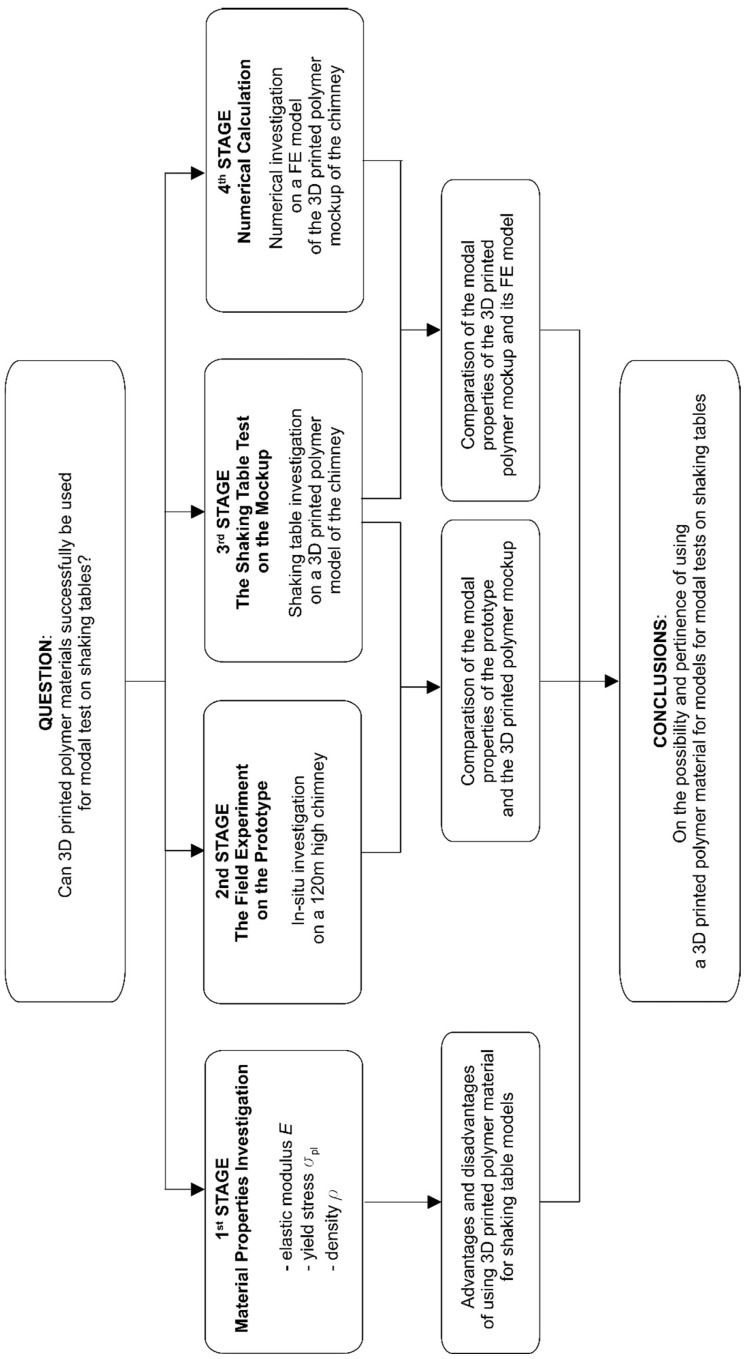
The general concept and the main stages of the research.

To assess the possibility and the pertinence of using 3D printed polymeric materials for models in modal tests on shaking tables, experimental and numerical works have been performed. The general concept of the research is presented in Figure 1.

In the first stage of the research, an investigation of the physical and mechanical properties of the 3D printed polymer material was carried out. The main properties, i.e., modulus of elasticity, yield stress, and mass density of the material were determined. Based on this part of the research, advantages and disadvantages of the 3D printed material, in the context of using it to create models for shaking tables, were recognized. 

In the second stage of the research, a field experiment on a reinforced concrete chimney 120 m high was executed. The experiment served to evaluate the modal properties (natural frequencies and damping ratios) of the prototype. In the third stage of the research, a shaking table test on a 3D printed polymer model of the chimney was performed. It allowed for the determination of the modal properties of the mockup. The comparison of the results obtained from the field experiment and the shaking table test allowed for the judgement of the advantages and disadvantages of using the 3D printed polymer model in the shaking table modal tests.

Finally, at the fourth stage of the research, the finite element (FE) model of the mockup was created, and the modal properties of the mockup were determined numerically. The goal of this stage was to match the numerical frequencies of the 3D printed polymer model with the measured values to meet the modal identification requirements. With the positive verification of the FE model, one can perform further numerical studies on determining the dynamic response of the 3D printed polymer model to dynamic excitations.

### 2.2. Fundamentals of the Theory of Similarity

Experimental research carried out on a shaking table requires the completion of an experimental model of a real structure (a prototype). The experimental model differs from the prototype in the size and type of material it is made of. Therefore, while preparing the experimental model, one should follow so-called similarity criteria [20,21,22]. Meeting these criteria guarantees that the behavior of the model during the shaking table tests will reflect the dynamic behavior of the prototype. Therefore, the essence of the application of the similarity criteria is the most accurate mapping of the geometry and material characteristics of the structure, as well as the boundary conditions and the external loads.

Compatibility between the object model and the prototype structure is ensured by carrying out the so-called dimensional analysis. In dimensional analysis, every physical variable $q_{1}$ can be expressed by a dimensionally homogenous equation:

(1) $$q_{1}=F\left(q_{2},\ldots ,q_{i},\ldots ,q_{n}\right)$$

where:

*n* denotes the number of variables describing the *q*_1_-phenomenon;
$q_{2},\ldots ,q_{i},\ldots ,q_{n}$ denote the physical quantities describing the *q*_1_-phenomenon.

According to the *π*-Buckingham theorem [23], every dimensionless variable $\pi_{1}$ can be presented in the form of a function depending on the independent dimensionless variables:

(2) $$\pi_{1}=F\left(\pi_{2},\ldots ,\pi_{i},\ldots ,\pi_{n-N}\right)$$

where:

$\pi_{2},\ldots ,\pi_{i},\ldots ,\pi_{n-N}$ denote the independent dimensionless variable;
*N* denotes the size of the dimensional base.

The experimental model completion, according to the π-Buckingham theorem, requires the selection of the dimensional base. Typically, the dimensional base consists of three independent variables on which the remaining parameters of the model will depend. In the case of an experimental model prepared for the needs of structural dynamics, which should accurately reflect these phenomena as dynamic characteristics, dynamic response to kinematic excitation, or resistance to earthquakes, Equation (1) can be written as:

(3) $$\sigma =F\left(d,E,\rho ,a,g,f,t,m,\sigma_{0}\right)$$

where:

*σ* is stress [Pa];
*d* is geometrical dimension [m];
*E* is Young modulus of material [Pa];
*ρ* is material density [kg/m^3^];
*a* is acceleration [m/s^2^];
*g* is gravity acceleration [m/s^2^];
*f* is natural frequency [Hz];
*t* is time [s];
*m* is mass [kg];
*σ_0_* is yield stress [Pa].

Moreover, the dimensionless form of Equation (3) can be introduced [24]:

(4) $$\frac{\sigma }{E}=F\left(\frac{a\cdot E}{d\cdot \rho },\frac{g\cdot E}{d\cdot \rho },\frac{f\cdot d\cdot \rho^{0.5}}{E^{0.5}},\frac{t\cdot E^{0.5}}{d\cdot \rho^{0.5}},\frac{m}{\rho \cdot d^{3}},\frac{\sigma_{0}}{E}\right)$$

In formula (4), the dimensional base consists of geometrical dimensions *d*, elasticity modulus *E*, and the material density *ρ*.

The dimensionless dependencies must be met for both the prototype and the experimental model. Therefore, the similarity scales take the form of Equation (5):

(5) $$S_{L}=\frac{L_{model}}{L_{prototype}}$$

where:

*S_L_* is the similarity criterion for variable *L;*
*L_model_* is the value of the variable *L* in the model;
*L_prototype_* is the value of the variable *L* in the prototype.

The similarity criterion should be defined and fulfilled for each relevant parameter of the phenomenon under study.

In practice, one of the following types of laboratory models is usually used: A True Strength Model, an Artificial Mass Model, or an Ignoring Gravity Model. Each of these model types has a different application and a different dimensional base and is defined by different similarity criteria (Table 1).

The first type of the model, i.e., the True Strength Model, is used when the stresses resulting from gravity forces are comparable to the stresses resulting from inertia forces occurring due to the dynamic loading of the structure. Therefore, neglecting gravity leads to unrealistic test results. The dimensional base in the True Strength Model consists of model size (length) *d*, elasticity *E*, and the gravity acceleration *g*. It should be noted that the gravity acceleration cannot be scaled; therefore, the gravity similarity parameter is equal to 1. In this case, the fulfilment of the similarity criteria related to the density of the material (see Table 1, column 3) is only possible if a material with significantly lower stiffness or a significantly higher density than the prototype material is used to build the model [23,25].

If it is not possible to fulfil the True Strength Model similarity criteria due to the material of the model properties, an Artificial Mass Model may be used. The dynamic characteristics of this model are enhanced by adding mass, which does not change the stiffness or strength of the model. This model will provide satisfactory results, provided that the mass is properly distributed and attached to the structure [23,25].

The last type of model, i.e., the Ignoring Gravity Model, can only be used when the stresses due to gravity are significantly smaller than the stresses induced by dynamic loads. Then, the similarity criterion related to the value of gravitational acceleration is neglected. The dimensional base in the Ignoring Gravity Model consists of model size (length) *d*, elasticity *E*, and the density *ρ*. It should be strongly pointed out that this model should be used only for the study of linear phenomena. In the case of non-linear effects, numerous errors and inaccuracies may appear due to the neglect of the gravity forces.

### 2.3. Structural Data of the Analyzed Chimney

**Figure 2:**
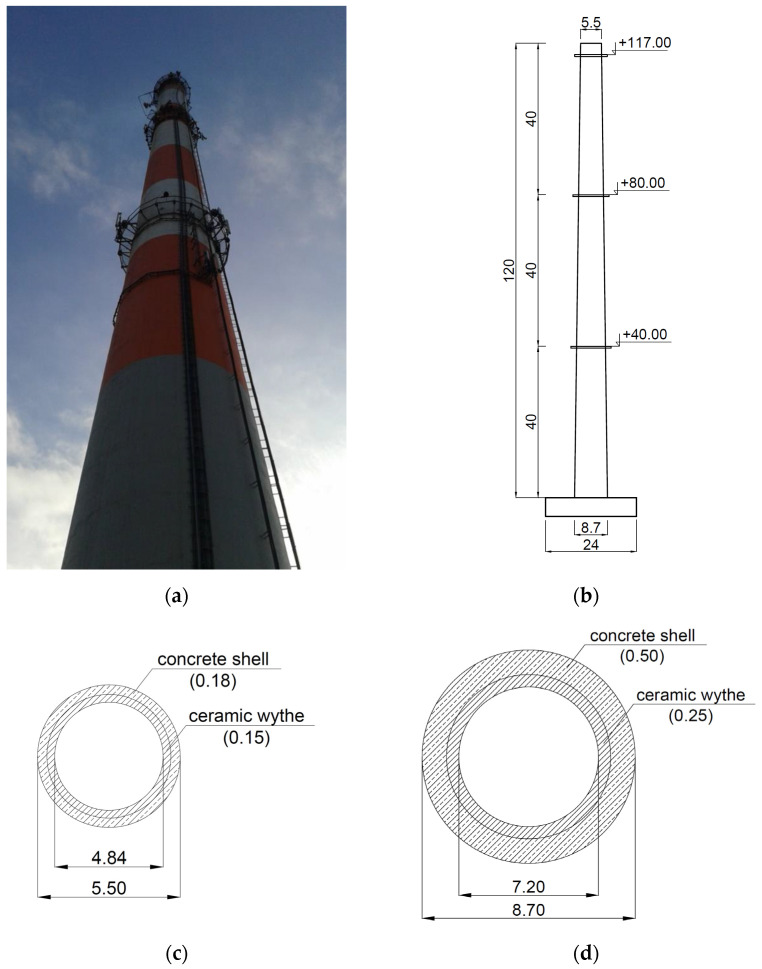
The analyzed chimney (the prototype): (**a**) The general view; (**b**) the main vertical dimensions [m]; (**c**) the dimensions of the cross-section at the top [m]; (**d**) the dimensions of the cross-section at the bottom [m].

A single-pipe reinforced concrete chimney with a ceramic flue gas lining, 120 m high, was selected as a prototype in the presented research. The object was erected in 1982 for the need of a municipal heating plant in Southern Poland. Due to a large number of chimneys of similar height and construction, the object appeared to be a representative example of this type of structure. Moreover, it was found that it had the best-preserved design and as-built documentation necessary for the proper realization of the model for the shaking table tests. The dimensions of the chimney are shown in Figure 2.

The chimney is founded on a reinforced circular foundation with a diameter of 24 m, made of the C16/20 concrete class [26]. The diameter of the chimney shaft is 8.7 m at the base and 5.5 m at the top. The thickness of the shaft is 50 cm at the base and 18 cm at the top. The shaft, made of the C16/20 concrete class, is reinforced with the St3S class steel [27]. The values of parameters used for the described chimney were: Elasticity modulus *E* = 29 GPa, the Poisson ratio *v* = 0.15, and the density *ρ* = 2500 kg/m^3^ for the C16/20 concrete, and *E* = 210 GPa, *v* = 0.3, and *ρ* = 7850 kg/m^3^ for the St3S steel. For the chimney shaft, the reinforcement ratio of 4% was taken into account by increasing the elasticity modulus of the concrete proportionally to the steel and concrete volume fraction. In this case, the elastic modulus was scaled up to the value of 35 GPa.

The ceramic lining of the chimney is made of bricks on cement-lime mortars with a density of 2000 kg/m^3^. The thickness of the lining is 25 cm in the bottom part of the chimney (up to 90 m above the ground) and 15 cm at the upper part of the object. There is an 8-cm thick thermal insulation between the lining and the reinforced concrete chimney shaft. The structure is equipped with three steel service galleries at the levels of +40, +80, and +117 m, and with external steel ladders along the entire height of the structure.

### 2.4. Experimental Set-Up for Determining the Properties of the 3D Printed Polymer Material

The assessment of the possibility of using experimental models made in 3D printing technology from polymers in modal tests on shaking tables must be based on correctly determined material parameters. In this work, it was decided to experimentally determine the physical and mechanical parameters of a PLA-IMPACT biodegradable polymer material and, based on them, assess the possibility of its use in 3D printed models proposed for testing on shaking tables.

The PLA-IMPACT material, with high resistance to cracking and fracture, is recommended for use in the case of elements that are exposed to work in difficult load conditions. The material is successfully used in the automotive and mechanical industries [28,29,30], as well as in bioengineering [31,32]. Therefore, it can be assumed that the PLA-IMPACT material will perform well under dynamic load conditions on a shaking table.

The following properties of the PLA-IMPACT material were determined experimentally by the elasticity modulus, the yield point, and the mass density.

**Figure 3:**
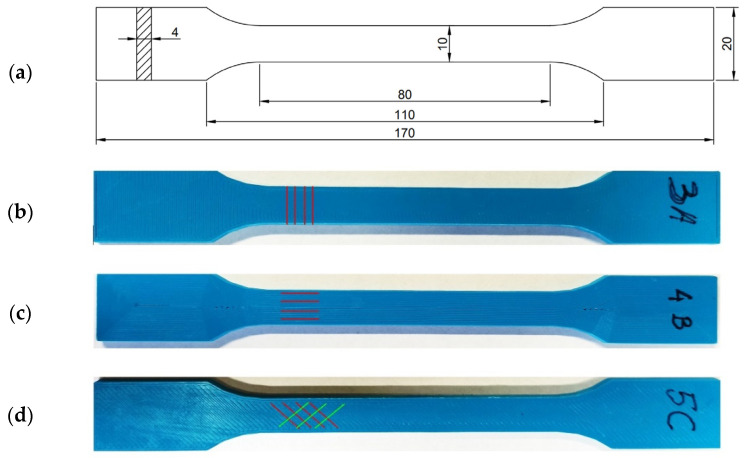
(**a**) The geometry of a PLA-IMPACT material specimen for a static tensile test and specimens printed with (**b**) transverse, (**c**) parallel, and (**d**) crosshatched raster.

Determination of the elasticity modulus and yield point of the PLA-IMPACT polymer material was carried out by recommendations in accordance with the Polish standards PN-EN ISO 527-1: 2020-01 [33] and PN-EN ISO 527-2: 2012 [34]. The elasticity modulus was determined in a static tensile test of dumbbell-shaped samples with dimensions shown in Figure 3. It is important that the sample was made by the method of continuous application of successive polymer layers, i.e., without any working breaks. This prevents the undesirable phenomenon of sample delamination during the test.

Due to the technological process of 3D printing, which consists of successively applying successive layers of material, printed models have a layered structure. Therefore, it should be expected that elements made in this technology may exhibit anisotropic features. As a consequence, it can lead to a significant differentiation of mechanical parameters of the printed element depending on the arrangement of the material fibers [35,36,37,38]. Therefore, the samples of the PLA-IMPACT material with the different arrangements of the layers had to be tested to determine the elasticity modulus and yield point for various lamination directions.

To examine the PLA-IMPACT material, 15 specimens were prepared with three different lamination configurations regarding the stretching direction (Figure 3): Five samples with the arrangement of layers in the transverse direction (samples with letter A), five samples with a parallel arrangement of layers (samples with letter B), and five samples with a crosshatched raster of layers at an angle of +/−45° (samples with letter C).

**Figure 4:**
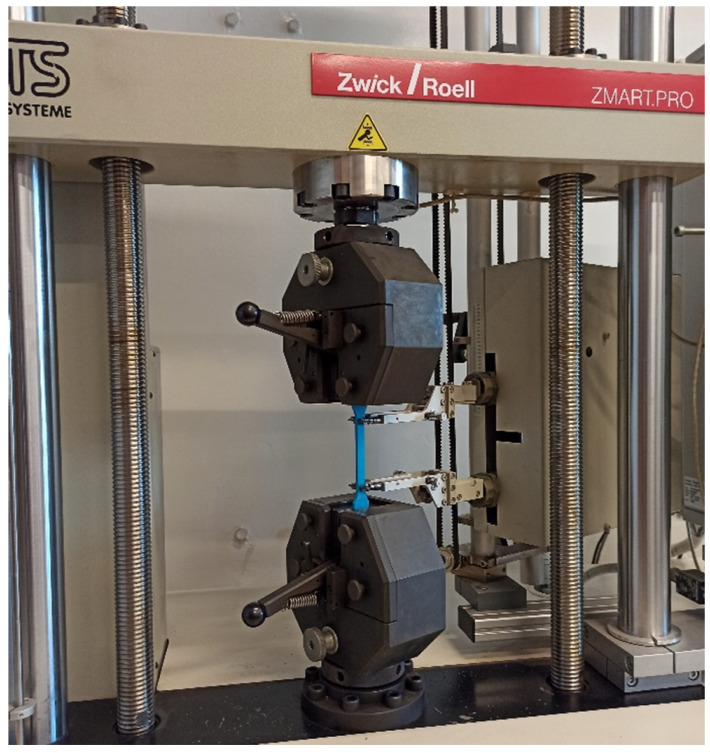
The Zwick Roell testing machine used for the static tensile test.

The tests served to determine the elasticity modulus of the PLA-IMPACT material were conducted with the use of the Zwick Roell testing machine (Figure 4). Subsequent specimens were placed in the jaws of the machine with an initial spacing of 115 mm. The distance between the extensometer grips (the length of the datum) was set at 75 mm.

Each sample was pre-stressed with a value of 0.5 MPa (a value which does not exceed 0.05% of the expected value of the elastic modulus) and then stretched at a constant rate of 1% strain per minute. During the measurement, the displacement of the machine head, the force, and the change in the length of the sample were recorded continuously. The test was carried out until the sample deformation exceeded 0.25%. The value of Young’s modulus was calculated based on formula (6):

(6) $$E=\frac{\sigma_{2}-\sigma_{1}}{\varepsilon_{2}-\varepsilon_{1}}$$

where *σ*_1_ and *σ*_2_ denote stress values under strain *ε*_1_ = 0.0005 and *ε*_2_ = 0.0025, respectively.

The yield point was also determined based on the static tensile test with the same parameters as those specified for determining the modulus of elasticity (measuring base of 75 mm, jaw space of 115 mm, initial stress of 0.5 MPa, test speed of 1% strain per minute). The stretching of each sample was continued until it broke. The value of the yield stress was taken as the value of the stress in the sample at the permanent relative deformation of 0.2%.

The material density was calculated based on the average weight of the sample and its volume. The porosity of the samples, which may result from the presence of holes and a gap between the individual fibers of the laminate, was neglected in the sample volume. This simplification was considered as acceptable in engineering applications.

### 2.5. Experimental Set-Up for the Field Investigation on the Modal Properties of the Chimney

**Figure 5:**
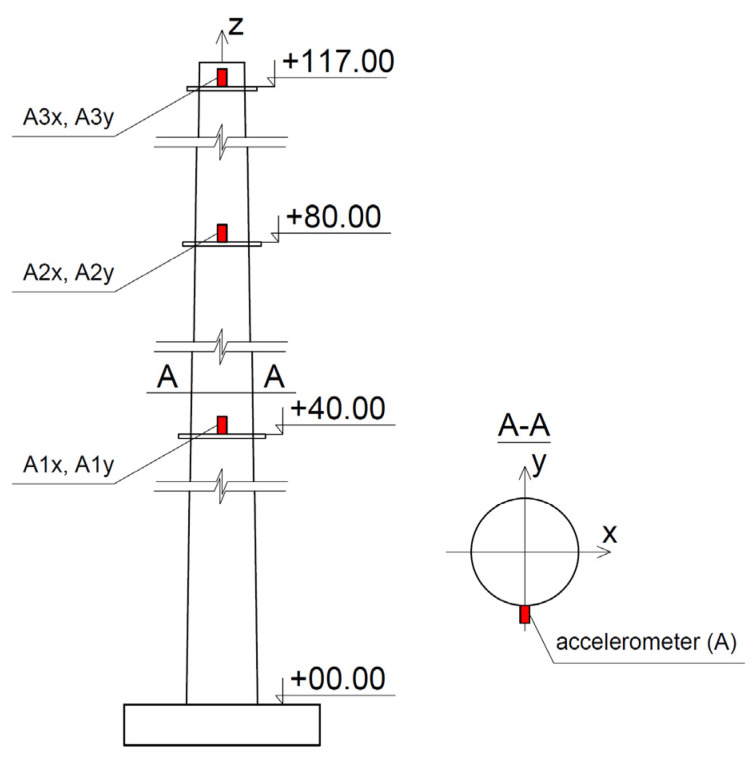
The arrangement of the accelerometers on the chimney shaft.

The dynamic characteristics of the chimney, i.e., natural frequencies and damping properties, were estimated through the field test. The experimental set-up consisted of three high sensitivity (10,000 mV/g) accelerometers (393B12 PCB Piezotronics) placed on the shaft of the chimney at a height of +40, +80, and +117 m. The accelerations were registered along the orthogonal axes X and Y of the chimney. The layout of the measuring points is shown in Figure 5. 

The measuring equipment also contained a signal amplifier, a Siemens LMS SCADAS Mobile recorder, and a laptop that served to preview the recorded vibrations. The frequency range of accelerometers was from 0.15 to 1000 Hz. All sensors were wire connected. The data sampling of the signal was 1024 Hz. The nominal deviation of the sensors and the signal amplifier was +/−5%, and the distortion of the signal for cables and the digital recorder was +/−1.5% and +/−0.05%, respectively. 

In the field experiment, the chimney vibration was induced by the movement of a group of four alpinists. The people, standing at the highest gallery (+117 m), were swaying rhythmically for 10 s. Then, after the people stopped swaying, the free vibration lasted about 50 s. The executed chimney vibration was recorded by the accelerometers placed on the levels of +40, +80, and +117 m.

### 2.6. Experimental Set-Up for the Shaking Table Test on the Modal Properties of the 3D Printed Polymer Model of the Chimney 

**Figure 6:**
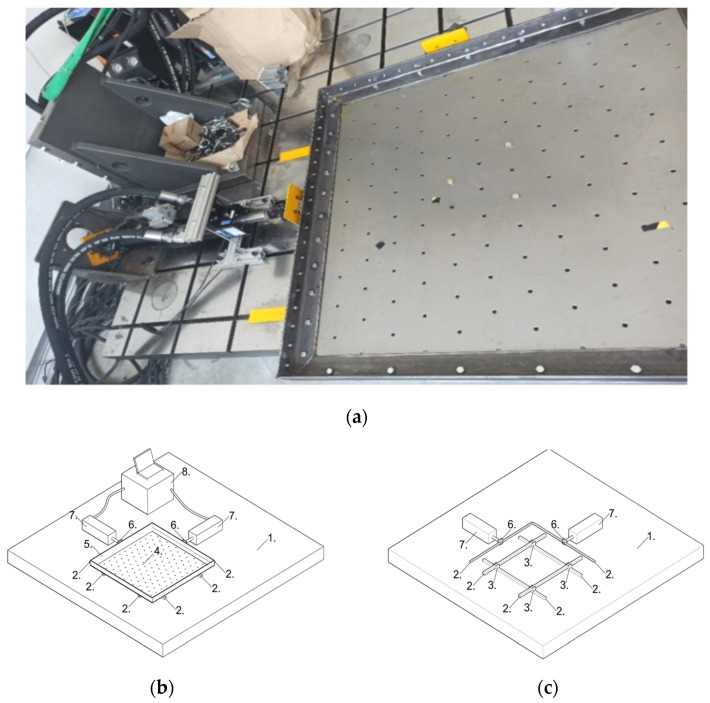
(**a**) The general view of the shaking table; (**b**) the scheme of the table; (**c**) the scheme of the support (1—the steel foundation; 2—the linear guides; 3—the carriages; 4—the operating platform; 5—the stiffening steel frame; 6—the actuator’s head on the linear carriage; 7—the hydraulic actuators; 8—the steering system).

The study of the dynamic characteristics of the 3D printed polymer model of the chimney was carried out on a shaking table constructed for the needs of the Chair of Mechanics of Structures and Materials of the Cracow University of Technology and the Cracow Branch of the ABB company. At the station, it is possible to generate horizontal vibrations (in two orthogonal directions) in a sinusoidal form, sweeps of frequency varying in the range of 0.2–100 Hz, and seismic vibrations. Depending on the frequency of a given excitation, the maximum amplitude ranges from 0.1 to 50 mm. The general view of the shaking table, its main components, and the foundation plate are shown in Figure 6.

The main component of the shaking table is a steel operating platform with regularly spaced holes, which serve for mounting experimental models. A model with a maximum base size of 1 × 1 m and a total weight of 300 kg can be placed on the platform.

The edges of the operating platform are stiffened with a frame made of steel sections (angles). The motion of the plate is possible due to hydraulic actuators. The actuators are rigidly attached to a steel support plate (foundation) and are steered by a control system integrated with the hydraulic pump. The heads of the actuators that move the platform are attached to two adjacent sides of the frame. They are connected to the frame through a system of carriages and linear guides. Using this system, it is possible to achieve the movement of the platform in two orthogonal directions.

### 2.7. Details of the Numerical Investigation on the 3D Printed Polymer Model of the Chimney

At the fourth stage of the research, according to the general concept of the study (see Section 2.1), a finite element (FE) model of the 3D printed polymer mockup used for shaking table tests was assembled, and natural frequencies and modes of vibrations of the mockup were numerically determined. With the positive verification of the FE model, one can perform further numerical research on determining the dynamic performance of the structure due to seismic excitation. Numerical investigation on natural frequencies and modes of vibrations of the model of the chimney was performed in Abaqus FEA [39] based on the Lanczos algorithm.

## 3. Results

### 3.1. Results of the Material Properties Investigation on the 3D Printed Polymer

At the first stage of the research, according to the general concept of the study (see Section 2.1), the physical and mechanical properties of the 3D printed polymer material were obtained through laboratory tests.

#### 3.1.1. The Elasticity Modulus of the 3D Printed PLA-IMPACT Material

**Figure 7:**
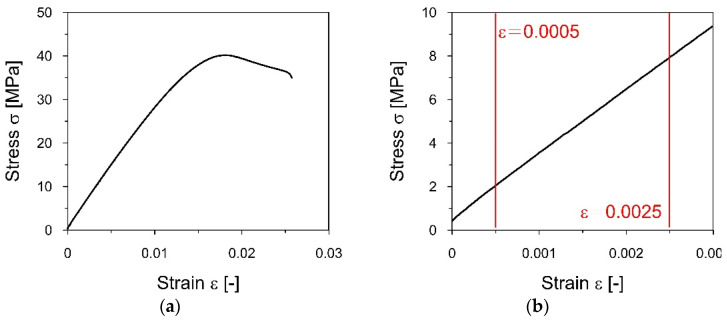
The strain−stress curve for the A1 specimen obtained from the tensile static test: (**a**) The whole registered experiment; (**b**) the detail of the curve used to determine the elasticity modulus.

The static tensile tests that served to determine the elasticity modulus of the PLA-IMPACT material were carried out according to the procedure described in Section 2.4. The Zwick Roell testing machine at a constant rate of 1% strain per minute was used. During the test, the displacement of the machine head, the force, and the change in the length of the sample were recorded. The example of the strain-stress curve obtained for sample 1A is shown in Figure 7. 

Based on formula (6), the value of the elastic modulus was determined for each specimen: A1–A5 samples with the arrangement of layers in the transverse direction, B1–B5 samples with a parallel arrangement of layers, and C1–C5 samples with a crosshatched raster of layers at an angle of +/−45°. The values obtained for particular specimens are summarized in Table 2. Based on the results in Table 2, it can be observed that the modulus of elasticity of the PLA-IMPACT material does not differ significantly for various directions of lamination.

#### 3.1.2. The Elastic Limit of the 3D Printed PLA-IMPACT Material

**Figure 8:**
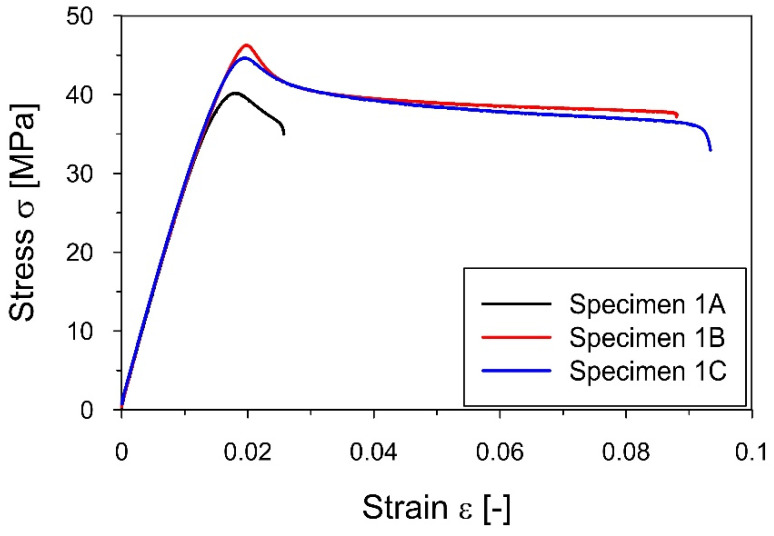
The strain−stress curves for A1, B1, and C1 specimens obtained from the tensile static test that served to determine the yield point of the PLA-IMPACT material.

The static tensile tests that served to determine the yield point of the PLA-IMPACT material were continued for each specimen until the sample broke. The yield point for a particular specimen was determined as the value of the stress at permanent deformation equal to 0.02%. The example of the strain-stress curves for specimens A1, B1, and C1 with different fiber arrangements is shown in Figure 8, whereas the yield stress values obtained for particular samples are summarized in Table 3.

The results presented in Table 3 demonstrate the differences in the values of the yield strength depending on the direction of lamination of the specimens. For the specimens with the longitudinal and the diagonal fiber arrangement, the yield stress was 41.9 and 40.5 MPa, respectively. The lowest value of 36.8 MPa was obtained for the specimens with transverse rastering, which was about 10% lower than the yield points obtained for the other lamination configurations. 

#### 3.1.3. The Mass Density of the 3D Printed PLA-IMPACT Material

The mass density was calculated based on the average weight of the sample and its volume. The average volume of a single sample was 9.6 cm^3^, and the average weight was 12.29 g. Therefore, the mass density of the PLA-IMPACT material was assessed to be 1.28 g/cm^3^.

### 3.2. Results of the Field Experiment on the Dynamic Properties of the Chimney

#### 3.2.1. Natural Frequencies of the Chimney Obtained from the Field Experiment

At the second stage of the research, according to the general concept of the study (see Section 2.1), two fundamental frequencies of the natural vibration of the chimney as well as the damping ratios accompanying these frequencies were assessed based on the field experiment. The set-up for the field experiment is specified in Section 2.5.

**Figure 9:**
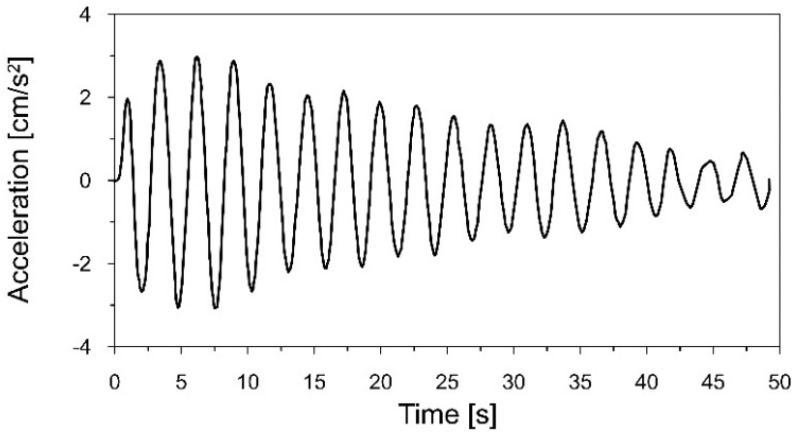
The acceleration−time history of the chimney vibration, induced by the movement of a group of people, registered at the level of the highest gallery (+117 m) in the X direction.

The acceleration-time history of the chimney vibration recorded at the level of the highest gallery (+117 m) in the X direction (see Figure 5) is shown in Figure 9.

**Figure 10:**
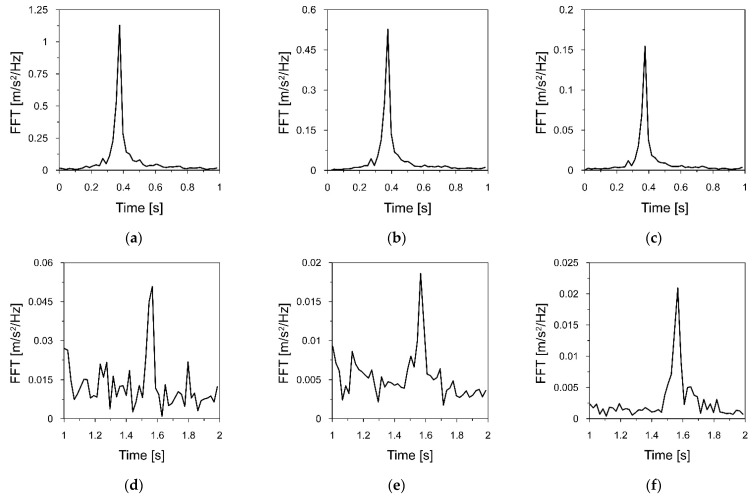
Fourier spectra of the acceleration−time histories that served to estimate the first natural frequency of the chimney vibration, registered at: (**a**) The highest gallery; (**b**) the middle gallery; (**c**) the lowest gallery, and the fragment of the Fourier spectra that served to estimate the second natural frequency, registered at: (**d**) The highest gallery; (**e**) the middle gallery; (**f**) the lowest gallery.

The Fourier spectra of the acceleration-time histories of the chimney vibrations registered at measurement points located at each gallery are presented in Figure 10.

The presented Fourier spectra show that the fundamental and the second natural frequencies of the chimney vibration were 0.365 and 1.570 Hz, respectively. It must be pointed out that the modes of vibrations, which accompanied the first and the second natural frequency, had forms typical for modes of cantilever natural vibrations.

#### 3.2.2. Damping Ratios of the Chimney Obtained from the Field Experiment

In the second stage of the research, damping ratios corresponding to the fundamental frequencies of the natural vibration of the chimney were also assessed based on the field experiment. The amplitudes of the chimney vibration indicate the decay, which appeared after the alpinists stopped swaying (see Figure 9). This part of the signal served for the evaluation of the damping properties of the chimney.

**Figure 11:**
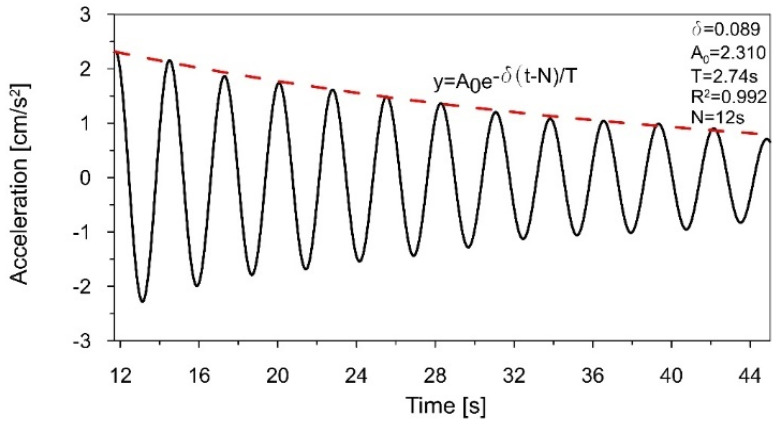
The fragment of the acceleration−time history, filtered around the first natural frequency *f*_1_ = 0.365 Hz that served to estimate the logarithmic decrement of damping *δ*_1_ = 0.089.

The acceleration-time history of the chimney vibration presented in Figure 9 was filtered around the first natural frequency *f*_1_ = 0.365 Hz with a narrow-band filter from 0.32 to 0.4 Hz. The sixth-order Butterworth filter was used, as it provides monotonic amplitude response without ripples in both passband and stopband, as well as the quick roll-off around the cutoff frequency. Based on the fragment of the acceleration-time history with the declining amplitudes, the logarithmic decrement of damping *δ*_1_ = 0.089 and the damping ratio ξ_1_ = 1.41%, corresponding to the first natural frequency, were calculated (Figure 11). The damping curve (red dashed line in Figure 11), based on the logarithmic decrement of damping, was calculated using the least squares method. The method allows for the fitting of a function to experimental data. The best-fitting curve is characterized by the smallest value of the sum of squared errors between the experimental values and the values from the fitted function. The measure of curve fitting is the determination coefficient R^2^. This coefficient takes values from 0 (in the case of no fitting of the curve to the experimental data) to 1 (in the case of a perfect fitting of the curve to the experimental data). In practice, if the value of the R^2^ coefficient is above 0.8–0.9, the fit can be considered as correct.

The same procedure was introduced to find the logarithmic decrement of damping and the damping ratio corresponding to the second natural frequency *f*_2_ = 1.570 Hz. The obtained values were *δ*_2_ = 0.132 and ξ_2_ = 2.10%, respectively. It is worth noticing that the obtained values of damping ratios, ξ_1_ = 1.41% and ξ_2_ = 2.10%, are in good agreement with data accessible in literature for high concrete chimneys [40,41].

### 3.3. Results of the Shaking Table Tests on the Dynamic Properties of the 3D Printed Model

#### 3.3.1. Adopted Similarity Criteria

In this research, the Ignoring Gravity Model was adopted (see Section 2.2). Therefore, during the dimensional analysis, the similarity criteria described in column 5 of Table 1 were taken into account. The model size (length) *d*, elasticity *E*, and the density *ρ* were adopted as the dimensional base. After transforming to the dimensionless form, accordingly to formula (4), the basic similarity criteria resulting from the adopted base were established as *S_d_* = 1/120, *S_E_* = 0.0842, and *S_ρ_* = 0.512. The remaining values of the similarity criteria are summarized in Table 4. In the chimney model, the similarity between the model and the prototype concerns structural elements, i.e., the chimney shaft. However, in the case of this structure, one should not forget about the key non-structural element, i.e., the ceramic lining, which significantly affects the dynamical characteristics of the object. The brick lining consists of segments about 10 m high, placed on special supports. There is an insulating material and an air gap between the lining and the chimney shaft. Therefore, the lining is disconnected from the chimney shaft. For this reason, the influence of the lining on the stiffness of the structure can be neglected. As this element does not add stiffness to the structure, it can be represented with a mass added to the structure. The mass size was determined based on the mass similarity criterion *S_m_*.

#### 3.3.2. The Model of the Chimney Made of the 3D Printed Polymer Material

**Figure 12:**
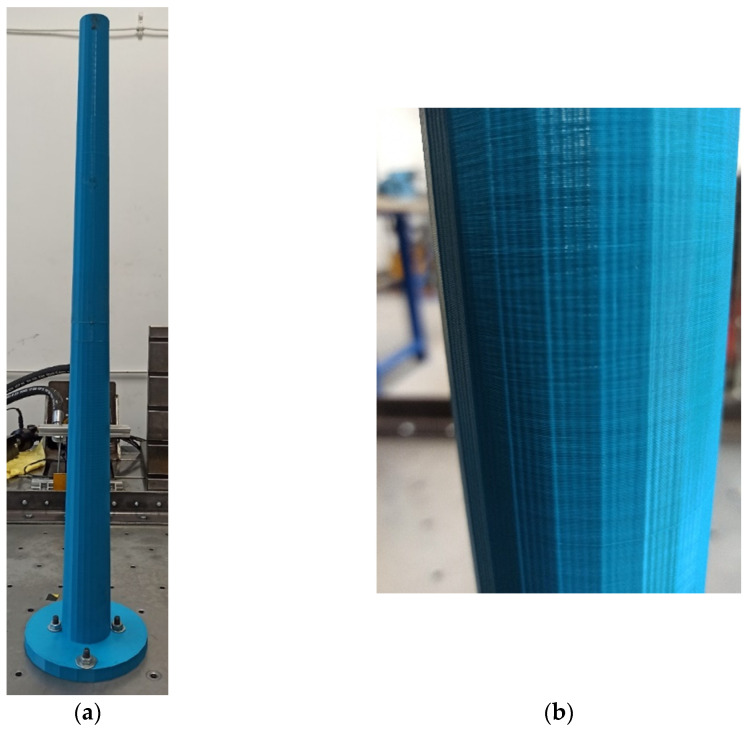
(**a**) The 3D printed polymer model of the chimney shaft made of PLA-IMPACT material; (**b**) the detail of the model with the lamination direction visible.

Once the similarity criteria were established, the model of the chimney made of the PLA-IMPACT material could be prepared. The model was printed with an industrial class machine Urbicum GX printer. The model was printed in a spiral manner with the horizontal direction of lamination. Figure 12a shows a 3D printed polymer model of the chimney shaft (1 m high), whereas Figure 12b presents a fragment of the model with the lamination exposed.

**Figure 13:**
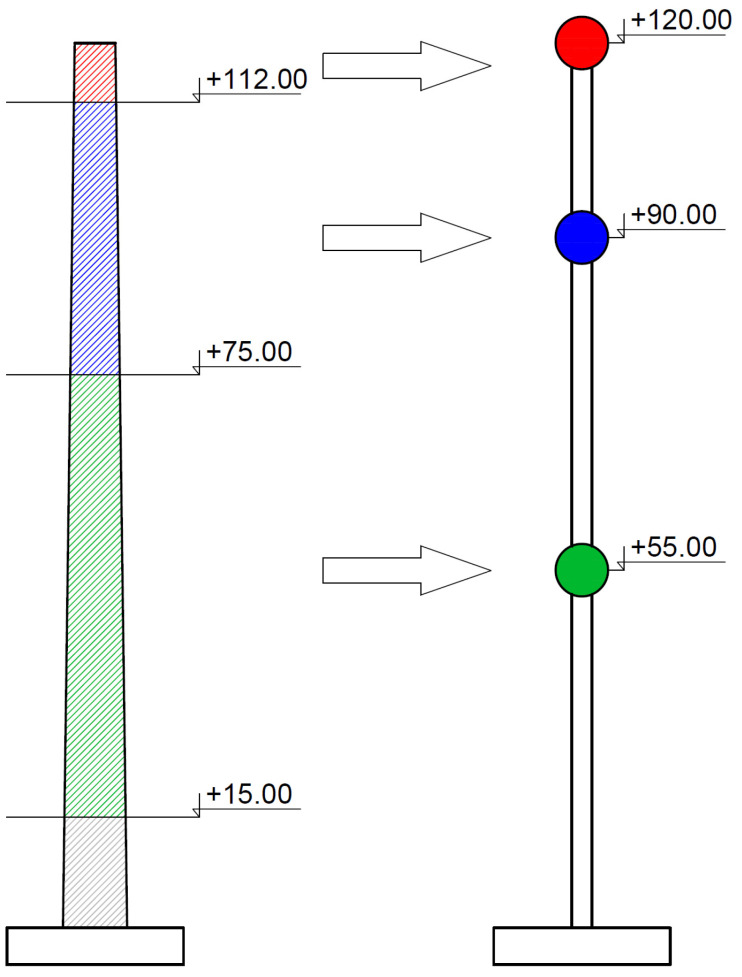
The scheme for determining additional masses and their arrangement.

The presented model imitated the concrete shaft of the chimney only. The mass resulting from the lining of the ceramic chimney has not yet been taken into account. Therefore, the model was equipped with additional masses to include the mass of the lining in the model. According to the dimensions presented in Section 2.3, the total mass of the lining was 1006.5 tons. This mass was divided into three parts of 40.5, 296, and 670 tons, located on the levels of 120, 90, and 55 m above the ground, respectively (at these levels, the brick lining is attached to the shaft of the prototype). The part of the lining mass, located up to 15 m above the ground, was attached to the ground level. The scheme for determining additional masses and their arrangement is presented in Figure 13. Then, based on the similarity criterion $S_{m}$ = 2.96·10^−7^ (see Table 4), the values of additional masses introduced into the model were determined as 12, 88, and 200 g. The locations of the additional masses were 1.0, 0.75, and 0.45 m above the ground. The mass attached to the ground level was not included in the model. The distribution of the lining mass and the arrangement of the additional masses of the model are summarized in Table 5.

**Figure 14:**
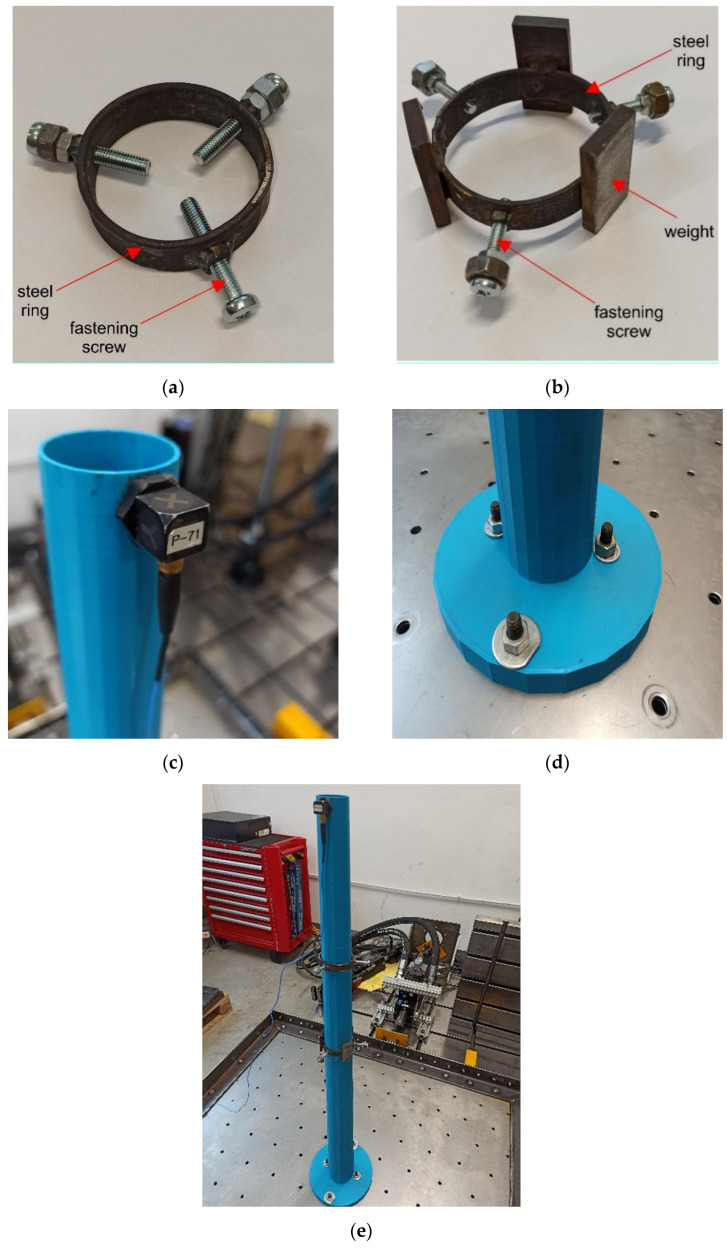
The main technical details of the model: (**a**) The clamp located on the level of 0.75 m; (**b**) the clamp located on the level of 0.45 m; (**c**) the accelerometer mounted at the top of the model; (**d**) the fixed support the model; (**e**) the final view of the chimney’s model, with the additional masses, fixed to the shaking table platform.

The main technical details of the model are presented in Figure 14. At the levels of 0.75 and 0.45 m above the ground, the masses were added in the form of steel clamps. Each clamp had the shape of a ring made of a steel sheet 3 cm wide and 2 mm thick. The diameter of each ring was matched to the diameter of the chimney model at the level of the ring. Therefore, the rings at the levels of 0.75 and 0.45 m had diameters of 55 and 60 mm, respectively.

Each ring was equipped with three screws, which after tightening, ensured a three-point connection of the ring with the outer surface of the chimney shaft model. This connection prevented the ring from moving during the shaking table tests, and at the same time, did not increase the circumferential stiffness of the shaft model. Due to the relatively large mass that had to be attached on the lowest level, additional steel plates were welded to the largest clamp. The clamps located on the levels of 0.75 and 0.45 m above the ground are presented in Figure 14a,b, respectively.

At the top level, a very small additional mass of 12 g had to be attached to the model. Moreover, a sensor measuring acceleration during the shaking table experiments had to be installed on this level. To meet both requirements, it was decided to install a lightweight sensor rather than the upper clamp. A ceramic-aluminium accelerometer (393B12 PCB Piezotronics) of a total mass of 12 g was used (the sensor and the washer masses were 7.4 and 4.6 g, respectively). The sensitivity of the accelerometer was 100 mV/g, the frequency range was from 0.5 to 5000 Hz, and it was wire connected to the measuring station. Mounting this accelerometer on the top of the model provided an additional mass that was exactly equal to the mass of the brick lining at the top level and fulfilled the mass similarity criterion. The accelerometer mounted at the top of the model is shown in Figure 14c.

The model was fixed to the shaking table with four screws, nuts, and lock washers. The screws connected the base of the model (a circular plate 2 cm thick with a diameter of 20 cm) and the operating platform. Two screws were located in the direction of excitation, and two in the direction perpendicular to the direction of excitation. The detail of the fixed support of the model is shown in Figure 14d. The mockup of the chimney, made of the 3D printed polymer material with the additional masses, fixed to the shaking table platform is presented in Figure 14e.

#### 3.3.3. Natural Frequencies of the 3D Printed Polymer Model Obtained from the Shaking Table Tests

At the third stage of the research, according to the general concept of the study (see Section 2.1), two fundamental frequencies of natural vibration of the 3D printed polymer model of the chimney were assessed based on the shaking table test. The experimental set-up for this test is described in Section 2.6. First, a linear sweep covering the range of 1–80 Hz was performed on the model. In the registered acceleration-time history, two significant signal amplifications were observed. They appeared between 20–30 and 80–90 s. At these moments of the linear sweep, the frequencies varied from 15 to 18 Hz and from 71 to 75 Hz, respectively. The observed signal amplifications indicated the first and second natural frequencies of the model. 

**Figure 15:**
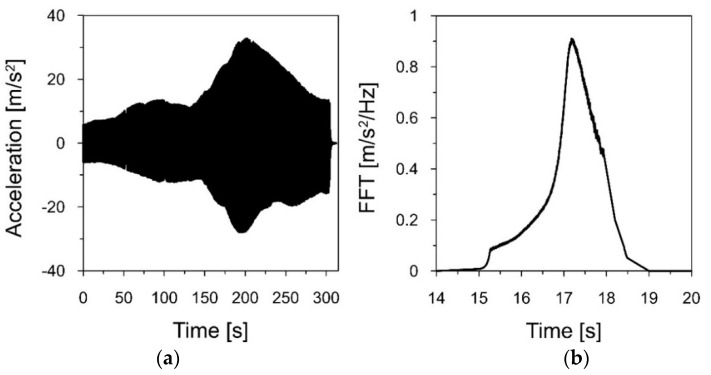
(**a**) The acceleration−time history of the model vibrations, caused by a linear sweep in the range of 15–18 Hz, registered at the top of the model; (**b**) the Fourier spectrum of the signal.

**Figure 16:**
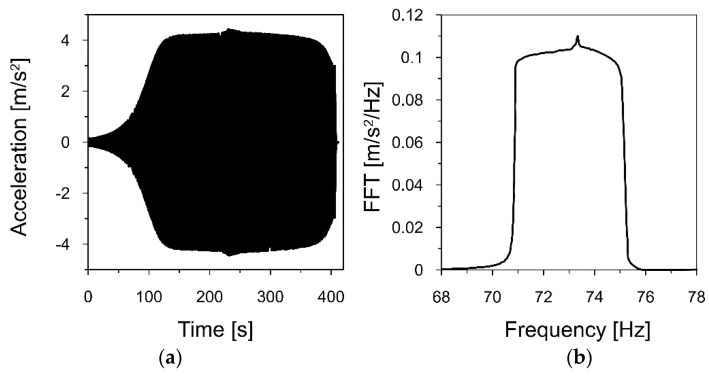
(**a**) The acceleration−time history of the model vibrations, caused by a linear sweep in the range of 71–75 Hz, registered at the top of the model; (**b**) the Fourier spectrum of the signal.

After estimating the approximate ranges for the first and second natural frequencies, linear sweeps were performed from 15 to 18 Hz and from 71 to 75 Hz, with a speed of 0.01 Hz/s and an amplitude of 0.2 mm. The acceleration-time history of the sweeps, registered by the accelerometer located at the top of the model, as well as the Fourier spectra of these signals are presented in Figure 15 and Figure 16, respectively.

The Fourier spectrum, presented in Figure 15b, shows the maximum value for the frequency of 17.184 Hz. Therefore, this is the first natural frequency *f*_1*m*_ of the 3D printed model. Taking into account the frequency similarity criterion $S_{f}=48.688$ (see Table 4), the first natural frequency of the chimney (the prototype) *f*_1*p*_ can be calculated as follows:

$$f_{1p}=\frac{f_{1m}}{S_{f}}=\frac{17.184}{48.688}=0.353\;\mathrm{Hz}$$

Then, the Fourier spectrum, demonstrated in Figure 16b, shows the maximum value for the frequency of 73.263 Hz. This indicates the second natural frequency *f*_2*m*_ of the model. Therefore, the second natural frequency of the chimney *f_2p_* takes a value of:

$$f_{2p}=\frac{f_{2m}}{S_{f}}=\frac{73.263}{48.688}=1.505\;\mathrm{Hz}$$

#### 3.3.4. Damping Ratios of the 3D Printed Polymer Model Obtained from the Shaking Table Tests

To determine the logarithmic decrement of damping corresponding to the first natural frequency of the 3D printed polymer model, the registered acceleration-time history of the model vibration in the range of 15–18 Hz (see Figure 15a) was filtered around the first frequency of 17.184 Hz, using a narrow-band filter with a width of 0.2 Hz (17.1–17.3 Hz).

**Figure 17:**
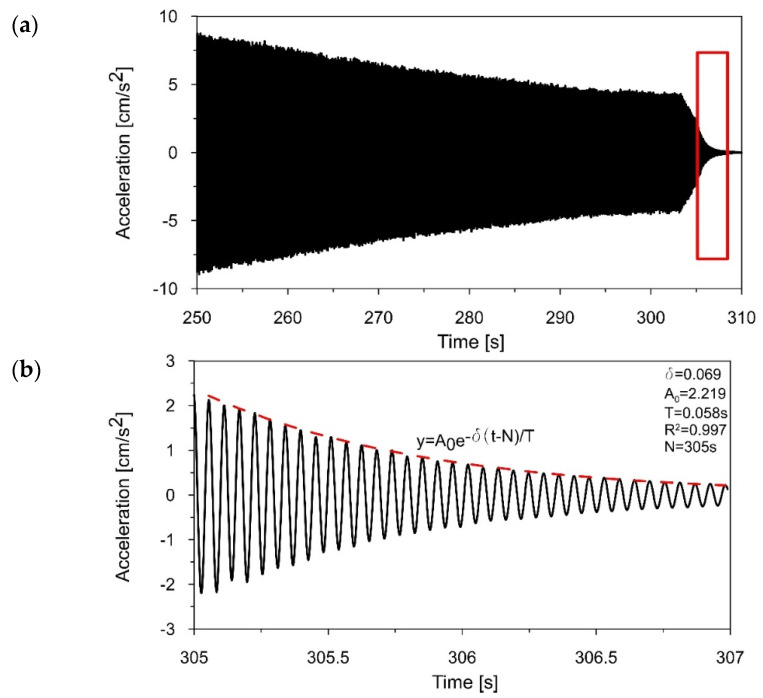
(**a**) The acceleration−time history filtered around *f_1_* = 17.184 Hz; (**b**) the decrease in amplitudes (305–307 s) that served to estimate the logarithmic decrement of damping *d*_1_ = 0.069.

Figure 17 presents the acceleration-time history of the filtered signal, showing the decrease in the vibration amplitude after the excitation stopped (305–307 s). Based on the decay of amplitudes, the logarithmic decrement of damping *δ*_1_ = 0.069 and the damping ratio ξ_1_ = 1.10%, corresponding to the first natural frequency, were obtained.

**Figure 18:**
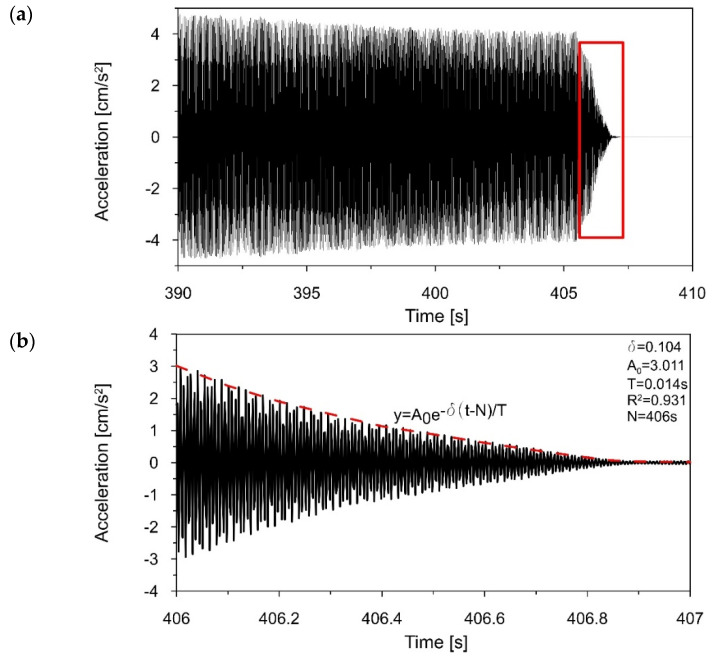
(**a**) The acceleration−time history filtered around *f*_2_ = 73.263 Hz, (**b**) the decrease in the amplitudes (406–407 s) that served to estimate the logarithmic decrement of damping *δ*_2_ = 0.104.

Then, to estimate the logarithmic decrement of damping corresponding to the second natural frequency, the recorded acceleration-time history of the model vibration in the range of 71–75 Hz (see Figure 16a) was filtered around the first frequency of 73.263 Hz, using a narrow-band filter with a width of 0.2 Hz (73.15–73.35 Hz). Figure 18 presents the acceleration-time history of the filtered signal, showing the decrease in the vibration amplitude (406–407 s). Based on the declining amplitudes, the logarithmic decrement of damping *δ*_2_ = 0.104 and the damping ratio ξ_2_ = 1.66%, corresponding to the second natural frequency, were calculated.

### 3.4. Results of the Numerical Calculation of the Dynamic Properties of the 3D Printed Model

#### 3.4.1. The FE Model of the 3D Printed Polymer Model of the Chimney

**Figure 19:**
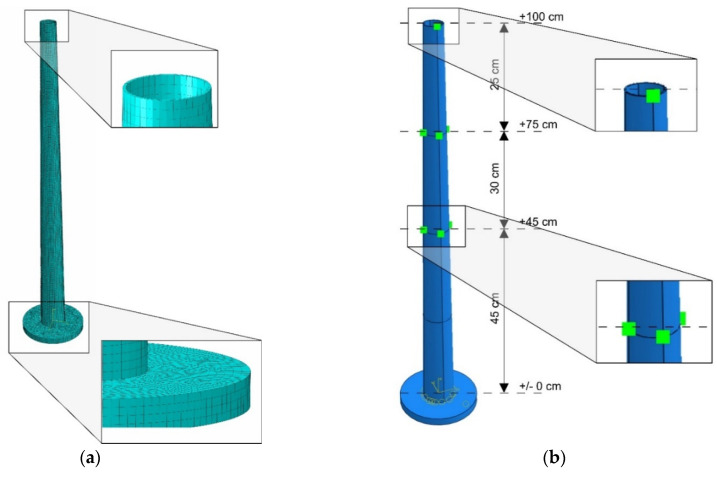
(**a**) The finite element model with the mesh details; (**b**) the additional masses assembly.

The FE model of the mockup is shown in Figure 19a. About 14,500 finite elements were incorporated into the model. The structural components of the chimney (the shaft and the foundation) were modeled with 8-node linear brick elements C3D8R (3 degrees of freedom in node, reduced integration with hourglass control).

An elastic material was assumed in the numerical model. The values of applied mechanical parameters were determined based on the experimental research (see Section 3.1): The elastic modulus *E* was 2.95 GPa and the mass density was *ρ* = 1280 kg/m^3^. The value of Poisson’s ratio, *v* = 0.33, was not determined experimentally, and its value was adopted based on literature [42]. Fixed support of the base of the structure was used as boundary conditions of the model.

To model the additional masses on three levels, lumped mass (point mass) elements were used. The highest mass was added at one point (similar to the sensor in the experiment), whereas the masses at the levels of +0.75 and +0.45 m above the ground were added to the structure at four points around the chimney circumference (Figure 19b). The sum of the masses at each level was equal to the mass determined based on the mass similarity criterion (see Table 5).

#### 3.4.2. Natural Frequencies of the 3D Printed Polymer Model Obtained from the Numerical Calculation

**Figure 20:**
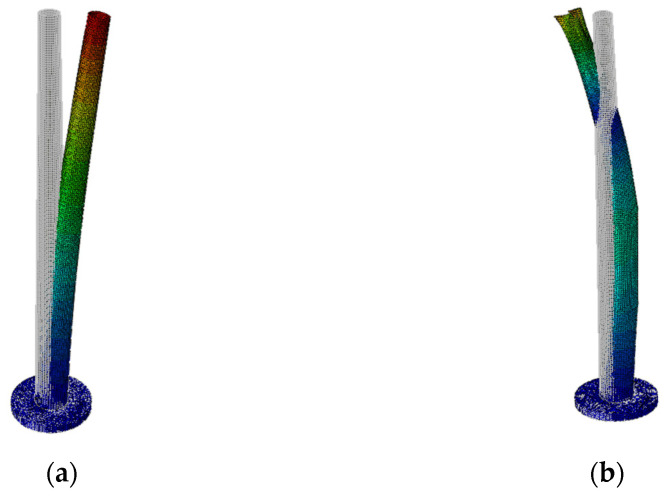
Basic modes of vibration obtained numerically: (**a**) First mode corresponding to frequency *f*_1_ = 0.360 Hz; (**b**) second mode corresponding to frequency *f*_2_ = 1.607 Hz.

Two basic frequencies were recognized numerically: *f*_1_ = 17.528 Hz and *f*_2_ = 78.242 Hz. After scaling by the similarity criterion, the following frequencies were obtained: *f*_1_ = 0.360 Hz and *f*_2_ = 1.607 Hz. The corresponding modes of vibration are presented in Figure 20.

## 4. Discussion

### 4.1. Comments on the Mechanical Properties of the 3D Printed Polymer in the Context of the Application of the Material to Models for Shaking Table Tests

Based on the mechanical properties determined from the material properties investigation, some advantages and disadvantages of using the PLA-IMPACT material to produce models for shaking tables can be discussed. Based on the results presented in Section 3.1, it can be observed that the modulus of elasticity of the PLA-IMPACT material does not differ significantly for various directions of lamination. This observation is of great importance when it comes to 3D printing models of tall structures, such as chimneys. The chimney model could only be printed in a spiral manner with the horizontal direction of lamination. No other direction of printing was technically possible. The presented results prove that the direction of lamination has no significant effect on the modulus of elasticity of the material. Therefore, in the calculations of the model printed from the PLA-IMPACT material, the values of the material’s modulus of elasticity can be used regardless of the direction of lamination.

The presented results indicated the differences in the value of the yield strength depending on the direction of lamination of the specimens. In the general case, when the ratio of the yield strength of the model’s material to the yield strength of the structure’s material is different from the ratio of their modulus of elasticity, the stresses obtained for the model working in the plastic zone will not meet the criterion resulting from the stress similarity scale. Therefore, it will not be possible to transfer stresses from model tests to real stresses in the structure. Therefore, when planning the research, it is important to ensure that the experimental model operates in the elastic range. From this point of view, the direction of the lamination of the model seems to be crucial.

### 4.2. Comments on the Natural Frequencies of the Chimney: The Field Experiment Results vs. the Shaking Table Test Results

The comparison of the natural frequencies of the chimney obtained from the field experiment and the shaking table test (scaled values) is presented in Table 6. The percentage errors, obtained for both frequencies, were equal to 2.77 and 3.82%, respectively. 

To assess the utility of any shaking table model, one should consider the discrepancy between the natural frequencies obtained from the field and the shaking table tests. If they are significantly above the acceptable limit, a tuning procedure has to be carried out. The most common tuning attempt of shaking table models consists of increasing the total mass of the mockup [43] to meet the requirement of the mass similarity relation. 

The agreement between the natural frequencies obtained from the field experiment and the shaking table test may be considered as reasonable, as compared, for example, to the results of modal tests presented in other works [14,15], since the differences are lower than 5%. Therefore, neither tuning nor any modifications of the 3D printed model were necessary to accomplish. The presented comparison of the natural frequencies is an important argument indicating the pertinence of using 3D printed materials to make models for shaking table tests.

### 4.3. Comments on the Damping Ratios Estimated from the Field Experiment and the Shaking Table Test

The damping ratios obtained from the field experiment for the chimney and from the shaking table test for the 3D printed polymer model are compared in Table 7.

Herein, the obtained damping ratios are worth commenting on. The accurate assessment of damping properties plays a crucial role in the evaluation of the dynamic performance of both the prototype and the model, under any dynamic loading or kinematic excitation. In the case where the material of the prototype and the material of the model are different, the criterion of similarity in terms of damping ratios is equal to S_ξ_ = ξ_model_/ξ_prototype_ [44]. For example, when the prototype was a three-story frame constructed of steel wide-flange beams and columns, and the model was plastic, fabricated using fused deposition modeling, the authors of the research estimated prototype damping as ξ_prototype_ = 0.117% and model damping as ξ_model_ = 1.5% [45].

In this study, from the performed field and the shaking table tests, it turned out that the criterion of similarity concerning damping was about 0.8 (the damping properties of the model were about 20% smaller than those of the prototype). Therefore, when planning shaking table tests with a model made of a 3D printed polymer material, one should experimentally determine the damping ratios of the 3D printed mockup. Otherwise, assuming that the similarity criterion value is equal to 1, and taking damping ratios of a prototype for further calculations of the dynamic performance of the 3D printed model, the assessment of the dynamic behavior of the model can be miscalculated. 

### 4.4. Comments on the Natural Frequencies of the 3D Printed Polymer Model: The Shaking Table Test Results vs. the Numerical Calculation Results

The comparison of the natural frequencies of the 3D printed polymer model obtained from the shaking table test and the numerical calculation is presented in Table 8. 

It is generally accepted in the literature that the similarity between numerical and experimental results, with respect to natural frequencies, is considered as satisfactory if the differences between them are less than 7% [46]. As shown in Table 8, the experimentally and numerically obtained natural frequencies of the 3D printed model indicate a good level of consistency, as the discrepancies are well below the acceptable limit. Therefore, the goal of the research, matching the numerical frequencies of the 3D printed polymer model with the measured values to meet the modal identification requirements, has been achieved.

However, when the discrepancy between the numerical and experimental natural frequencies is significantly above the acceptable limit, a tuning of the numerical model has to be performed. The first tunning attempt of the FE model of the mockup, according to the authors’ experience, should concern the location of additional masses in the FE model. The location of the additional mass in the numerical model must strictly correspond to the location of the masses in the 3D printed model. The ring-to-shaft connection in the mockup is achieved by three screws (see Section 3.3.2). The points of contact of the screws with the model determine the positions of additional masses. However, the lower edge of the ring may adhere to the shaft of the mockup, which indicates that the actual location of the additional mass is different from the assumed one. The mislocation of additional masses in the FE model may result in unacceptable inconsistency of experimental and numerical frequencies.

## 5. Conclusions

In this article, the possibility and the pertinence of using 3D printed polymeric materials for models in modal tests on shaking tables have been recognized. Four stages of the research have been linked: The material properties investigation, the field experiment on the modal properties of the reinforced concrete chimney, the shaking table test on the modal properties of the 3D printed polymer model of the chimney, scaled according to the similarity criteria, and the numerical calculations of the FE model of the 3D printed mockup.

The obtained results allow for the formulation of some important conclusions concerning the possibility and the pertinence of using 3D printed polymer materials for models in modal tests on shaking tables. Some basic advantages and disadvantages of using these 3D printed polymeric models were also identified:

1. The investigation of the properties of 3D printed polymer materials revealed that the direction of lamination had no significant effect on the modulus of elasticity of the material. This is a great benefit, especially when printing models of tall structures, such as chimneys, which for technical reasons could only be printed in a spiral manner with the horizontal direction of lamination. Moreover, in further dynamic calculations of the 3D printed polymer model, the values of the material’s modulus of elasticity can be used regardless of the direction of lamination.
2. The investigation of the properties of 3D printed polymer materials also proved that the yield strength depended on the direction of lamination of the specimens. The transfer of stresses from shaking table tests with 3D printed polymer models to real stresses in a concrete structure is possible only when the model works in the elastic range. Stresses obtained for the 3D printed polymer model working in the plastic zone will not meet the criterion resulting from the stress similarity scale. This is a certain disadvantage of these models. Therefore, when performing shaking table tests with 3D printed polymer models, it is important to ensure that the model works in the elastic range. From this point of view, the direction of the lamination of the model seems to be crucial.
3. The modal properties of the chimney were assessed through the field experiment and the shaking table tests. It turned out that the natural frequencies of the chimney and the model showed good compatibility. This is a substantial argument indicating the pertinence of using 3D printed polymer materials to create models for shaking table tests. Moreover, it occurred from those experiments that the criterion of similarity concerning damping ratios (S_ξ_ = ξ_model_/ξ_prototype_) was equal to about 0.8, which indicates that the damping properties of the model were about 20% lower than those of the prototype. Assuming that the most common similarity criterion value is equal to 1 (as if the prototype and the model are made of the same material) in further dynamic analyses, this can lead to the improper assessment of the 3D printed model performance.
4. To perform dynamic analyses in parallel on a mockup and its FE model, the numerical model has to be positively verified as far as modal properties are concerned. In the presented case, the similarity of the natural frequencies of the 3D printed polymer model obtained from the shaking table test and the numerical calculation may be considered as satisfactory. However, sometimes discrepancies between experimental and numerical frequencies may occur to be far from the acceptable limit. Then, a tuning of the FE model must be performed. To the authors’ knowledge, inaccuracies between modal properties of 3D printed mockups and their FE models may appear mostly due to the incorrect introduction of additional masses into FE models.

The presented analysis may be supportive in answering the question of whether traditional models (made of the same material as prototypes) used in shaking table tests are still the best solution, or whether innovative 3D printed polymer models can be a better choice, in regard to the assessment of the modal properties and the dynamic performance of structures.

## Figures and Tables

**Table 1:** Similarity criteria for different types of dynamic models.

Scaling Parameter	Similarity Criterion	Model Type		
		True Strength Model	Artificial Mass Model	Ignoring Gravity Model
Length *d*	$S_{d}$	$S_{d}$	$S_{d}$	$S_{d}$
Elasticity *E*	$S_{E}$	$S_{E}$	$S_{E}$	$S_{E}$
Density ρ	$S_{\rho }$	$S_{d}^{-1}\cdot S_{E}$	neglected	$S_{\rho }$
Time *t*	$S_{t}$	$S_{d}^{0.5}$	$S_{d}^{0.5}$	$S_{d}\cdot S_{\rho }^{0.5}\cdot S_{E}^{-0.5}$
Frequency *f*	$S_{f}$	$S_{d}^{-0.5}$	$S_{d}^{-0.5}$	$S_{d}^{-1}\cdot S_{\rho }^{-0.5}\cdot S_{E}^{0.5}$
Acceleration *a*	$S_{a}$	1	1	$S_{d}^{-1}\cdot S_{\rho }^{-1}\cdot S_{E}$
Gravity *g*	$S_{g}$	1	1	neglected
Strain *ε*	$S_{\varepsilon }$	1	1	1
Stress *σ*	$S_{\sigma }$	$S_{E}$	$S_{E}$	$S_{E}$
Mass *m*	$S_{m}$	$S_{d}^{2}\cdot S_{E}$	neglected	$S_{d}^{3}\cdot S_{\rho }$

**Table 2:** The values of the elasticity modulus obtained for particular specimens from the tensile static tests.

Specimen Type A	Elastic Modulus [GPa]	Specimen Type B	Elastic Modulus [GPa]	Specimen Type C	Elastic Modulus [GPa]
A1	2.95	B1	2.90	C1	2.94
A2	2.93	B2	2.94	C2	2.90
A3	2.89	B3	3.03	C3	2.96
A4	2.96	B4	3.01	C4	2.95
A5	2.90	B5	2.92	C5	2.94
E_mean_ [GPa]	2.93		2.96		2.94
Standard deviation	0.02		0.05		0.02

**Table 3:** The values of yield stress obtained for particular specimens from the tensile static test.

Specimen Type A	σ_pl_ [MPa]	Specimen Type B	σ_pl_ [MPa]	Specimen Type C	σ_pl_ [MPa]
A1	36.62	B1	41.03	C1	41.21
A2	37.22	B2	42.21	C2	39.79
A3	38.16	B3	43.68	C3	40.11
A4	36.98	B4	41.79	C4	40.80
A5	35.02	B5	40.88	C5	40.47
σ_pl_ [GPa]	36.80		41.92		40.48
Standard deviation	1.03		1.01		0.50

**Table 4:** Similarity criteria determined for the analyzed chimney.

Similarity Criterion		Criterion Value
Length	$S_{d}$	0.00833
Elasticity	$S_{E}$	0.0842
Density	$S_{\rho }$	0.512
Time	$S_{T}$	0.0205
Frequency	$S_{f}$	48.688
Acceleration	$S_{a}$	19.754
Gravity	$S_{g}$	neglected
Strain	$S_{\varepsilon }$	1
Stress	$S_{\sigma }$	0.0842
Mass	$S_{m}$	2.96·10^−7^

**Table 5:** The distribution of the brick lining mass of the chimney (the prototype), and the arrangement of the additional masses of the model.

The Distribution of the Brick Lining Mass of the Prototype		The Arrangement of the Additional Masses of the Model	
Mass Location above the Ground [m]	Mass Attached at Particular Levels [ton]	Mass Location above the Ground [m]	Mass Attached at Particular Levels [g]
120	40.5	1	12
90	296	0.75	88
55	670	0.45	200

**Table 6:** The natural frequencies of the chimney obtained from the field experiment and the shaking table test.

Frequency Number	Frequency of the Chimney [Hz] Obtained from:		Error [%]
	Field Experiment	Shaking Table Test (Scaled)	
1	0.36	0.35	2.77
2	1.57	1.51	3.82

**Table 7:** Damping ratios estimated for the prototype from the field experiment and for the 3D printed polymer model from the shaking table test.

Mode Number	Damping Ratio [%]		Model/Prototype Damping Ratio
	Prototype	3D Printed Model	
1	1.42	1.10	0.77
2	2.10	1.66	0.79

**Table 8:** The comparison of the natural frequencies of the 3D printed polymer model obtained from the shaking table test and the numerical calculation.

Mode Number	Frequency of the 3D Printed Polymer Model [Hz] Obtained from:		Error [%]
	Shaking Table Test	Numerical Calculation	
1	17.184	17.528	1.98
2	73.263	78.242	6.78

## Data Availability

Not applicable.
